# UPDATE IN GEROSCIENCE FOR NONBIOLOGISTS

**DOI:** 10.1093/geroni/igac059.1344

**Published:** 2022-12-20

**Authors:** George Kuchel, Steven Austad

## Abstract

Geroscience began as a result of discoveries made by biologists in aging cells, simple organisms and animal models. Such advances, the discovery of strategies for modifying rate of aging, and appreciation of aging as a shared risk factor for chronic diseases led to the Geroscience Hypothesis. It states that interventions modifying aging biology can slow its progression, resulting in the prevention or delay of onset of multiple diseases. However, for these discoveries to truly impact human health, nonbiologists – scientists in other disciplines and clinicians, must also engage in geroscience. An obstacle to a wider engagement by disciplines and GSA members other than basic biologists in geroscience has been the fact that varied fields engaged in aging issues tend to use language (jargon), methods and scientific approach that can make their work less accessible and understandable to other disciplines. To that end, speakers have been selected not only for their scientific contributions, but also for their ability to overcome such barriers. Steve Austad (UAB) will discuss the range of animal models and experimental approaches that led to the Geroscience Hypothesis. Anne Newman (U Pittsburgh) will review evidence that these findings are relevant to and might be translatable to humans. John Newman (UCSF/Buck) will seek to frame these considerations in terms of broader societal discussions regarding aging while providing a framework by which nonbiologists may critically evaluate discoveries and actual products claiming benefits in terms of aging. (Sponsored by Nathan Shock Coordinating Center U24AG056053; Geroscience Education and Training Network R25AG073119).

